# Considerations raised during the regulatory and ethics review of platform clinical trials in infectious diseases

**DOI:** 10.1016/j.conctc.2026.101633

**Published:** 2026-03-26

**Authors:** Amos J. de Jong, Denise van Hout, Janneke D.M. Verberk, Marjolein P.M. Hensgens, Alike W. van der Velden, Janneke H.H.M. van de Wijgert, Marc J.M. Bonten, Lennie P.G. Derde

**Affiliations:** a Julius Center for Health Sciences and Primary Care, University Medical Center Utrecht, Utrecht, the Netherlands; b European Clinical Research Alliance on Infectious Diseases, Utrecht, the Netherlands; c Department of Infectious Diseases, University Medical Centre Utrecht, the Netherlands; d Intensive Care Center, University Medical Center Utrecht, Utrecht, the Netherlands

**Keywords:** Regulatory science, Clinical trial authorization, Platform trials, Adaptive designs, Trial innovation

## Abstract

**Background:**

Platform trials can address more than one clinical research question simultaneously and allow for the addition or removal of arms over time. The scientific and operational complexity of platform trials may give rise to specific challenges. In this study, we evaluate the considerations raised during the regulatory and ethics review of five infectious disease platform trials.

**Methods:**

Considerations raised during the review of five platform trials (ECRAID-Prime, RECOVERY, RECLAIM, REMAP-CAP, and SNAP) between 31 January 2022 and 28 March 2025, and sponsor responses were analyzed using content analysis. A single consideration could include multiple questions or remarks, and these were each treated as individual comments in the analysis.

**Results:**

Findings were categorized under four main themes: (i) clinical trial application, (ii) recruitment and informed consent, (iii) participant safety and data protection, and (iv) methodology. We identified 1218 comments, of which 93 (7.6%) specifically related to the platform trial design. Related to the methodology, regulators frequently requested more details about interim analyses, including triggers, correction for multiplicity, and stopping rules.

**Conclusions:**

Less than 10% of the comments was directly related to the platform trial design. These were usually resolved by providing additional explanation. No comments were identified in areas where feedback was anticipated by the research team, such as response adaptive randomization or confidentiality of interim results. Effective evaluation of platform trials requires sponsors to highlight platform trial-specific features in clinical trial applications, and for regulatory bodies to be adequately prepared to evaluate them.

## 1 Background

Platform trials are a type of randomized controlled trial addressing more than one clinical question simultaneously within a single master protocol, with the potential to add or drop interventions and incorporate new questions as needed [[1], [2], [3]]. They can continue if there are patients with the disease and outstanding research questions, provided that practical issues such as funding and study personnel are available. Additionally, platform trials can include adaptive features, such as preplanned adaptations to eligibility criteria, endpoints, and randomization ratios [4,5].

Due to these advantages, platform trials are increasingly being conducted [6]. However, given their scientific and operational complexity, appropriate measures are required to ensure safety oversight, data integrity, and participant understanding [[7], [8], [9], [10], [11]]. These elements are assessed by national competent authorities (NCAs) and medical research ethics committees (MRECs) during the clinical trial authorization process [12]. Despite the growing use of platform trials, little is known about how platform trial-specific features are evaluated during regulatory and ethics review and what concerns are considered important. Understanding this can improve platform trial design, protocol development, clinical trial applications, and the regulatory review process. Therefore, this study systematically analyzed considerations and sponsor responses from the regulatory review of five infectious disease platform trials.

## 2 Methods

### 2.1 Study design

In this qualitative study, we analyzed the considerations of European NCAs and MRECs that were raised during the regulatory and ethics review (hereafter referred to as “regulatory review”) and the trial sponsors’ responses all platform trials that were managed by the European Clinical Research Alliance on Infectious Diseases (Ecraid, www.ecraid.eu). We analyzed all regulatory reviews performed under the Clinical Trials Regulation (CTR, EU 536/2014) and in the UK between 31 January 2022, when the Clinical Trials Information System (CTIS) became operational and the CTR became applicable in the EU [13], and 28 March 2025. The Consolidated Criteria for Reporting Qualitative Research (COREQ) checklist was used to report on the methodology and results [14].

### 2.2 Platform trials

Five investigator-initiated platform trials evaluating interventions for infectious diseases, conducted in Europe, were included: ECRAID-Prime, RECOVERY, RECLAIM, REMAP-CAP, and SNAP (Appendix A). Reviews from EU countries were included. As Ecraid also manages REMAP-CAP and ECRAID-Prime in the United Kingdom (UK), those regulatory reviews were included. We included both initial clinical trial applications and any subsequent substantial modifications. For REMAP-CAP, the initial submission occurred under the Clinical Trials Directive (2001/20/EC). Therefore, we included the CTR transition submission and the first substantial modification submitted under the new regulation. All included platform trials have a modular protocol structure, with a master (or core) protocol describing the generic governing structure and design, supplemented by appendices describing individual treatments or treatment domains [2]. Additional appendices may include details on statistical analyses, region- and/or country-specific provisions, or specific study procedures (e.g., sampling, or observational registry components). SNAP and RECOVERY were authorized as low-intervention clinical trials, which was justified based on the fact that these trials evaluate authorized investigational medicinal products (IMPs) within the terms of the marketing authorizations.

### 2.3 Data collection

Data were extracted from CTIS and, for the UK reviews, requested from the respective study teams. Requests for information (RFIs, which contain multiple regulatory considerations and sponsor responses), assessment reports, cover letters, and pre-clinical trial application advice (for ECRAID-Prime) were analyzed. Documents that were not written in English or Dutch were translated using Google Translate (https://translate.google.com/). Basic clinical trial characteristics were obtained from CTIS and the most recent trial protocol.

### 2.4 Data analysis

Data were analyzed using content analysis using NVivo (version 15) [15]. Document text was broken down into segments and labelled to capture the meaning of each segment [15]. The content analysis was both deductive, using predetermined categories, and inductive. Deductive coding was informed by Articles 5 to 7 of the CTR and predetermined categories included: completeness of the dossier, therapeutic and public health benefits, risks and inconveniences for participants, compliance with manufacturing and import of IMP, compliance with labelling, requirements informed consent, arrangements for recruitment of participants, compliance with the General Data Protection Regulation, and investigator (site) suitability. Inductive analyses allowed for a sufficient level of granularity in the analysis and when reporting the results. We used the numbering provided in the RFIs to determine the number of considerations per trial. A single consideration could contain multiple questions, suggestions, or remarks, which were divided into individual comments for analysis. Comments pertaining to platform trials were coded accordingly. After coding the considerations and responses, the codes were grouped into meaningful categories and themes. Documents for one trial (SNAP) were coded in duplicate to agree on a preliminary code book. The other trials were coded by one researcher and checked by a second researcher. Disagreements were resolved by consensus. To refine the code book, findings were discussed iteratively within the research team. All themes including relevant examples pertaining to platform trials were described. Key findings were furthermore summarized in recommendations.

## 3 Results

### 3.1 Platform trial characteristics and themes

**Fig. 1:**
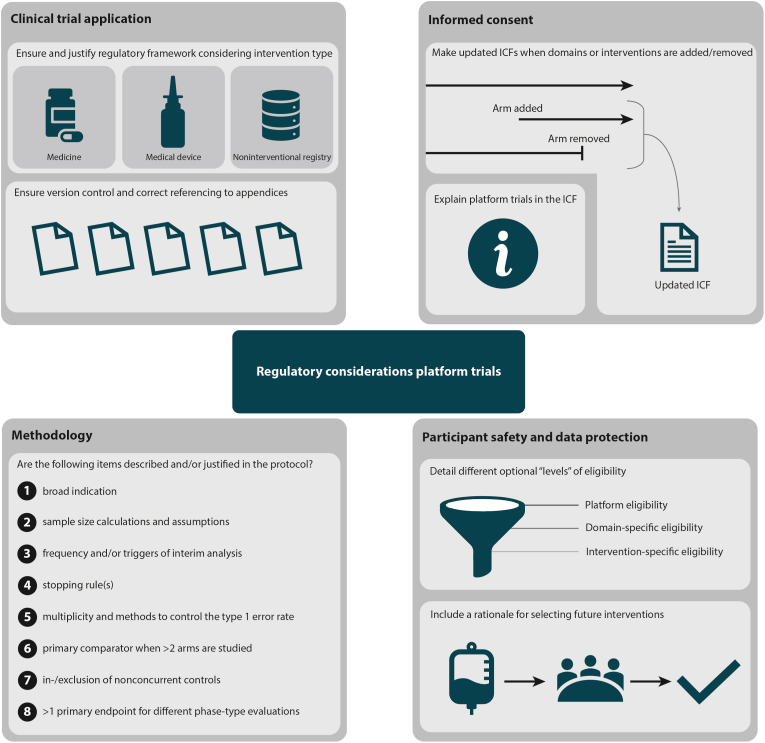
Key recommendations for the submission of a platform trial, categorized by theme. ICF, informed consent form.

Four of the five trials evaluated authorized IMP, although not necessarily within the authorized indication, and across different medical conditions. The number of countries included, ranged from 1 to 14 per trial (Table 1). In total, 901 considerations were identified: ECRAID-Prime (n = 506), RECOVERY (n = 135), REMAP-CAP (n = 115), SNAP (n = 103), and RECLAIM (n = 42). These considerations contained 1218 individual comments, of which 93 (7.6%) pertained to platform trials. Findings were categorized under four main themes: (i) clinical trial application, (ii) recruitment and informed consent, (iii) participant safety and data protection, and (iv) methodology (Table 2). All themes including relevant examples related to platform trials are described below and summarized in Fig. 1. Findings, including those applicable to trials in general (e.g., recruitment and consent procedures, data protection, and data collection), were summarized in practice-based recommendations (Appendix B).

**Table 1:** Characteristics of included platform trials.

Characteristic	Platform trial				
Trial acronym	ECRAID-Prime	SNAP	RECLAIM	REMAP-CAP	RECOVERY
EUCT number(s)	2022-501707-27-00 2022-501707-27-01	2023-503582-35-00	2024-511580-28-00 2024-511580-28-01 2024-511580-28-02	2023-507889-89-00	2023-507441-29-00
Sponsor	UMCU	UMCU	UMCU	UMCU	Oxford University
Trial phase	2/3	4	3	2/3	4
Medical condition	COVID-19 and COVID-like-illness	*Staphylococcus aureus* bacteremia	Post-acute sequelae of SARS-CoV-2 infection	Respiratory tract infection	Pneumonia
Low-intervention trial	Decided per compound	Yes	No	No	Yes
Status investigational medicinal products^1^	Unauthorized	Authorized	Authorized	Authorized	Authorized
Number of sites in the EU and UK^2^	38	37	1	163	63
Number of countries^2^	7	3	1	14	9
First patient first visit	9 October 2024	26 October 2023	27 February 2025	11 April 2016	22 March 2024

Characteristics registered in CTIS on 28 March 2025. ^1^ Of the authorized products, the studied indications do not all fall within the scope of the marketing authorization. ^2^ EU countries and the UK (for REMAP-CAP and ECRAID-Prime) managed by Ecraid. CTIS, clinical trial information system; EU, European Union; IMP, investigational medicinal product; UMCU, University Medical Center Utrecht.

**Table 2:** Comments categorized per theme.

Theme, sub-theme, topic	Platform trial-specific comments N = 93 (%)	Generic (not platform trial-specific) comments N = 1125 (%)
**1. Clinical trial application**	**32 (34.4)**	**656 (58.3)**
*1a. Regulatory management*	*6 (6.5)*	*11 (1.0)*
Out of scope CTR	6 (6.5)	5 (0.4)
Process regulatory review	0 (0.0)	6 (0.5)
*1b.Submission requirements*	*26 (28.0)*	*645 (57.3)*
Completeness submission	4 (4.3)	274 (24.4)
Clinical Trial Information System requirements	1 (1.1)	37 (3.3)
Document formatting	5 (5.4)	126 (11.2)
Generic clarifications	16 (17.2)	208 (18.5)
**2. Recruitment and informed consent**	**19 (20.4)**	**258 (22.9)**
*2a.Arrangements for the recruitment of trial participants*	*0 (0.0)*	*24 (2.1)*
Decentralized trial recruitment	0 (0.0)	5 (0.4)
Recruitment details	0 (0.0)	8 (0.7)
Rewarding or compensating trial participants	0 (0.0)	11 (1.0)
*2b.Requirements for informed consent*	*19 (20.4)*	*234 (20.8)*
Additional explanation & participant understanding	9 (9.7)	218 (19.4)
Informed consent approach	10 (10.8)	16 (14.0)
**3. Participant safety and data protection**	**6 (6.5)**	**170 (15.1)**
Data protection	1 (1.1)	46 (4.1)
Investigational medicinal product	1 (1.1)	55 (4.9)
Safety management	4 (4.3)	69 (6.1)
**4. Methodology**	**36 (38.7)**	**41 (3.6)**
Appropriateness of platform design	5 (5.4)	0 (0.0)
Blinding and comparisons	2 (2.2)	7 (0.6)
Data collection procedure	0 (0.0)	9 (0.8)
Endpoints	1 (1.1)	7 (0.6)
Indication	3 (3.2)	5 (0.4)
Statistical approaches	25 (26.9)	13 (1.2)

CTR, clinical trials regulation (EU 536/2014).

### 3.2 Clinical trial application

#### 3.2.1 Regulatory management

Six regulatory management comments were specific to platform trials, which suggested that not all parts of the trial were within the CTR scope (Table 2). The CTR applies to evaluations of IMP. One of the interventions in ECRAID-Prime is a nitric oxide (NO) nasal spray, which is registered as a CE-marked class 1 medical device but was submitted as an IMP. The sponsor justified this decision by stating the spray is considered to have a pharmaceutical effect and that future interventions in the platform trial may include IMPs. Assessors requested proof of CE certification because the study would otherwise be classified as a combination study (i.e., a trial evaluating both a medical device and IMP) or medical device performance study, which would fall under the Medical Device Regulation rather than the CTR.

In SNAP, the noninterventional registry component was considered outside the scope of the CTR according to assessors in Germany. Assessors requested that the sponsor follow national legal requirements for observational studies. The sponsor eventually submitted the registry study separately to local German MRECs. In contrast, assessors in the Netherlands and Sweden accepted a combined review of both the interventional and noninterventional components of SNAP.

#### 3.2.2 Submission requirements

Twenty-six comments relating to submission requirements were platform trial-specific (Table 2). These included generic clarifications (n = 16), document formatting requests (n = 5), requests to upload additional documents (n = 4), and naming of appendices in CTIS (n = 1). Regarding the completeness of the submission, for example, the assessors mentioned that future additions or removal of trial arms, requires the reassessment of new insurance certificates. Additionally, assessors required an explanation of the surplus of participants on the insurance certificate compared to the expected number of participants, due to the theoretical perpetual nature of platform trials. Assessors emphasized that trial documents must be consistently updated and aligned with prior changes, especially in complex modular protocols with multiple, interconnected appendices that require clear naming, proper referencing, and version control (Appendix B).

### 3.3 Informed consent

Nineteen comments relating to informed consent were specific to platform trials. Ten of these requested clarifications of the informed consent approach and nine requested revisions of the informed consent forms (ICFs). Primarily, sponsors were reminded to draft new ICFs when interventions are removed or added, and that these are subject to regulatory approval. Assessors furthermore requested an explanation of what a platform trial is in the ICF and whether participants can select which of the locally available treatment domains they want to participate in.

### 3.4 Participant safety and data protection

Six platform trial-specific comments related to participant safety and data protection (Table 2): safety management (n = 4), IMP selection (n = 1), and data protection (n = 1). Regarding safety management, for example, assessors noted that it is essential to clearly describe the participation of vulnerable groups, which may vary across different domains. More generally, domain-specific eligibility criteria can differ, which resulted in some confusion during the assessments. As an example, participation of children may be allowed (and described in the master protocol), but they may still be excluded from specific domains, interventions, or in certain countries. In one instance, regarding the selection of IMP, the assessors requested a rationale for the selection of future (currently unknown) IMPs for inclusion in the platform trial. In another instance, assessors asked for clarification of the duration of data retention in the context of the platform trial. The sponsor replied that data will be retained for 25 years after the end of the entire platform trial or after the closure of one specific domain, whichever comes first.

### 3.5 Methodology

Most platform trial-specific comments related to the methodology (Table 2), including the inclusion of two primary endpoints (n = 1), comparators (n = 2), indication under study (n = 3), appropriateness of the platform design (n = 5), and statistical approaches (n = 25) (Table 2).

#### 3.5.1 Indication, comparators, and endpoints

Platform trials may study broad indications and questions were raised on the specific indications under study. For example, in the case of ECRAID-Prime, diagnostics to differentiate between viral etiologies may not be available to general practitioners. For this reason, “COVID-like illness” was included in the trial indication. The assessors agreed with the broad indication but recommended including only IMPs with a known general antiviral activity. For the SNAP trial, it was requested to prespecify subgroup analyses because the study population is heterogeneous. When multiple interventions are evaluated, it may not always be apparent what the primary comparison is. In turn, the sponsor may be requested to specify this, as occurred for ECRAID-Prime, which compared NO to saline nasal spray. Because saline may have some antiviral activity, a “no intervention” usual care arm was added, resulting in two primary comparisons: NO vs saline nasal spray (blinded) and saline nasal spray vs usual care (open). Similarly, a master protocol may specify multiple primary objectives, depending on the phase type (i.e., phase 2 or phase 3) evaluation. For ECRAID-Prime, assessors requested a justification for this approach.

#### 3.5.2 Appropriateness of platform design

For ECRAID-Prime and RECLAIM, assessors requested a justification for the platform design, rather than a conventional randomized trial or group sequential design, when only two arms are compared. While assessors of the SNAP trial acknowledged the benefits of a platform design, they mentioned that it is necessary to have a robust infrastructure for performing high-quality interim analyses with complete and accurate data to leverage the benefits of the platform design.

#### 3.5.3 Statistical approaches

These are indeed funders of the trials. Three additional trial funders are cited: the Flu Lab (RECOVERY), Stichting Long Covid (RECLAIM), and the University Medical Center Utrecht (SNAP). The funding statement in the article proof is correct. Specifically, sample size calculations and the expected number of eligible patients to adjudicate feasibility were requested. Details of the model parameters and assumptions used to conduct the simulations were requested, including the definition of “power” when evaluating multiple interventions. That is, whether power was defined as the probability that at least one null hypothesis is rejected or the null hypotheses for all IMPs within a domain.

For ECRAID-Prime, RECOVERY, and SNAP, the sponsor was requested to provide additional information on the adjustment for covariates. Strategies to consider covariates in the analyses as proposed by the sponsors included stratified randomization, adjustment for covariates with existing imbalances, and prespecified subgroup analyses. Platform trials enable the addition or removal of interventions over time, allowing the use of historical controls. Assessors of the SNAP trial asked whether nonconcurrent controls would be included when new domains are added later. The sponsor explained that nonconcurrent controls are included in the primary analysis model and that time trend adjustment is applied to enhance the precision of the estimates. They added that a sensitivity analysis with concurrent controls may be conducted.

Assessors of ECRAID-Prime, RECOVERY, and SNAP requested more Information on the (interim) analyses, including the analysis outcomes, the statistical model, analysis triggers, correction for multiplicity, predefined rules to stop a specific arm, and the involvement of a data safety monitoring board (DSMB). For example, the sponsor of ECRAID-Prime explained that efficacy and futility thresholds were determined through simulations to ensure adherence to frequentist properties of power and error rate. In the same RFI document, however, assessors questioned the incorporation of clinically relevant information, noting that the superiority threshold should be based on a minimal clinically relevant difference (in hazard ratio), rather than solely derived from simulations. This appeared to reflect a misunderstanding of the sponsor's approach, in which “simulations” referred to statistical modelling used to set thresholds that still incorporate clinically relevant assumptions. The sponsor clarified that the parameters used in the simulations were chosen based on clinical relevance and information from previous studies.

Assessors of the RECOVERY trial mentioned that it should be clear when an intervention is considered efficacious or futile to avoid researchers making subjective decisions on stopping an arm, but that this would also reduce the flexibility of the adaptive platform trial. Therefore, the assessors agreed that no stopping rules were prespecified, but required that this should be mentioned in the protocol. For the SNAP trial, assessors considered the Bayesian framework and multiple stopping rules appropriate due to the potential for early stopping, the ability to borrow information, and the study of interactions. However, they mentioned that formal checking of the simulations was not possible without access to the simulation syntax due to the complexity of the analyses. It appeared that a review of the syntax did not preclude the authorization of the trial.

## 4 Discussion

In this study, we evaluated the comments raised by NCAs and MRECs during the regulatory review of five infectious disease platform trials. We found that 7.6% of the comments were platform trial-specific. Platform trial-specific comments were usually resolved by providing additional explanation or justification. Most platform trial-specific comments focused on statistical approaches, particularly interim analysis procedures. For platform trials, key information being spread across multiple trial documents may lead to clarifying questions and impede evaluation. An additional challenge can arise when platform trials include a noninterventional registry or evaluate medical devices, governed by distinct regulatory frameworks. Overall, approximately half of the comments related to submission requirements. This warrants a submission system and submission requirements that are straightforward and user-friendly, and requires sponsors to clearly communicate essential information and comply with submission requirements.

### 4.1 Methodology and statistics

Most platform trial-specific comments related to the methodology, in particular statistical approaches. We found that interim analysis procedures should be described in detail, including triggers, the role of the DSMB, and the rationale for stopping criteria thresholds. This algins with the experience of the ACTIV platform trials, where regulators expressed concerns about the adequacy of DSMB oversight during clinical trial conduct [8]. Interestingly, in contrast, assessors of the RECOVERY trial mentioned that not specifying stopping criteria beforehand allows for more flexibility and accepted this strategy. Although the need for strict type 1 error control in the context of trials supporting regulatory decision-making has been reiterated in various guidelines [4,16,17], evidence from the RECOVERY trial has previously been used to support the extensions of tocilizumab and casirivimab/imdevimab indications to patients with (severe) COVID-19 [18,19].

Platform trials can incorporate various adaptive design elements. For example, REMAP-CAP and SNAP can make use of response-adaptive randomization (RAR), which allows for adjustments to randomization ratios in proportion to the probability of success based on adaptive analyses [20,21]. There are different interpretations of the concept of equipoise related to RAR rooted in the distinction between theoretical equipoise, which assumes complete uncertainty about which treatment is better, and clinical equipoise, which requires sufficient uncertainty to ethically justify randomization [22,23]. In the current study, we did not identify any comments related to RAR, potentially making it a more theoretical than a practical concern. Similarly, the complexity of the platform design may affect participant autonomy [23,24], but few comments requested additional information detailing the platform design for participants. Additionally, it was not specified what information should be added to the ICF in this regard. Other potential concerns related to the platform design, including confidentiality of interim results, were not identified in the analyzed comments [4,16,25]. Furthermore, one consideration, only raised in the assessment of the SNAP trial, related to the use of nonconcurrent controls. The trial protocols may have been clear in these regards and considered acceptable by the assessors. Nonetheless, with the rise of trials using a platform design, there is a need to equip assessors with the necessary skills to evaluate these trials effectively.

### 4.2 Combined studies and European harmonization

Platform trials may evaluate multiple interventions and allow for the addition and removal of interventions during their lifetime, including medicinal products, medical devices, and diagnostics. In the EU, these evaluations are governed by different regulatory frameworks. Similarly, observational studies fall outside the scope of the CTR when medicinal products are evaluated in the context of routine care without additional diagnostic or monitoring procedures (Article 2, Regulation (EU) 2014/536). Registries, however, can be an integral part of a clinical trial. Registries are particularly valuable for assessing aspects such as the representativeness of the trial population, monitoring long-term outcomes, and providing contextual data that strengthen interpretation of trial results. From both scientific and operational standpoints, it is preferred for all components of a clinical study to be evaluated by the same assessors and submitted through a single platform. In practice, however, there is heterogeneity in how combined studies of medicinal products and medical devices are approached across EU countries, as acknowledged in the COMBINE project's results [26]. Expanding such initiatives to explicitly include registries as part of clinical trials could support more consistent and streamlined evaluation.

Efforts to further consolidate assessors’ comments across EU countries and implementing risk-proportional assessments – an area that has received insufficient attention in the context of investigator-initiated low-intervention trials – would help optimize reviews and timelines [[27], [28], [29]]. Specifically in the context of platform trials that evaluate authorized IMPs, risk-proportional approaches to authorization – for example through the low-interventional clinical trial category – should be considered to simplify trial procedures, such as the use of authorized commercial labeling and dispensing following routine care processes [28]. Additionally, from an implementation perspective, certain CTIS requirements may be particularly restrictive for platform trials, such as the inability to process multiple substantial modifications concurrently. All platform trials included in this study were submitted as a single clinical trial with multiple subprotocols, reflecting their shared governance, safety, and methodological framework – allowing for a consolidated review. For some master protocols, it may be possible to submit subprotocols as separate clinical trials, providing greater flexibility when frequent modifications are anticipated [7,17]. However, this is generally not feasible for multifactorial platform designs and would introduce fragmented regulatory and ethics assessments of shared elements that are part of a single overarching trial.

### 4.3 Strengths, limitations, and recommendations

This is the first study to systematically evaluate the comments of MRECs and NCAs raised during the regulatory review of platform trials. The current study included documentation of five platform trials in the field of infectious diseases that are managed by a single clinical research network (i.e., Ecraid). The platform trials were all publicly sponsored, investigator-initiated trials. We only included regulatory reviews for EU countries and the UK that were performed since the CTR became applicable. This meant that the initial application of REMAP-CAP was not included, as it was conducted under the Clinical Trials Directive 2001/20/EC. All these factors may impact the generalizability of the study findings to other therapeutic areas, geographical regions, and commercially sponsored trials.

Based on our findings, several key lessons emerge for optimizing the review of platform trials. Sponsors should ensure that submissions of dossiers are complete and well-structured. Specifically, for platform trials, they should clearly communicate procedures and documents that differ from those in conventional trials (e.g., insurance arrangements in perpetual trials, the modular protocol structure, and eligibility criteria in relation to future domains) to facilitate an appropriate regulatory evaluation. Likewise, regulators should consider integrating the evaluation of combined studies (i.e., trials evaluating both medicinal products and medical devices, or trials with a registry component) to allow for in-depth review by the same committee, thereby supporting consistent and efficient decision-making.

## 5 Conclusions

Regulatory reviews of platform trials generated relatively few platform trial-specific comments (7.6%). In these comments, assessors requested further clarification. The comments did not result in substantial protocol changes. Nonetheless, certain features, such as modular protocols, combined interventional/observational components, and complex statistical methods, are recurrent sources of questions. Differences between countries in evaluating trials with a registry component underscore the need for greater harmonization across the EU. No comments were received relating to key regulatory aspects specific to platform trials, such as RAR and confidentiality of interim results, highlighting the need for targeted training of assessors and clearer guidance for sponsors to ensure platform trials are adequately reported and reviewed. Overall, the results of this study emphasize that strengthening reporting on methodological aspects that differ from conventional trials, particularly statistical methods, is key to ensuring consistent and effective regulatory evaluation.

## CRediT authorship contribution statement

**Amos J. de Jong:** Writing – original draft, Investigation, Formal analysis, Data curation, Conceptualization. **Denise van Hout:** Writing – review & editing, Investigation, Formal analysis, Data curation, Conceptualization. **Janneke D.M. Verberk:** Writing – review & editing, Investigation, Conceptualization. **Marjolein P.M. Hensgens:** Writing – review & editing, Investigation, Conceptualization. **Alike W. van der Velden:** Writing – review & editing, Investigation. **Janneke H.H.M. van de Wijgert:** Writing – review & editing, Investigation. **Marc J.M. Bonten:** Writing – review & editing, Investigation, Conceptualization. **Lennie P.G. Derde:** Writing – review & editing, Investigation, Conceptualization.

## Ethics approval and consent to participate

This study does not fall under the scope of the Dutch Medical Research Involving Human Subjects Act (WMO). It therefore does not require approval from an accredited MREC in the Netherlands. An independent quality check was performed within the UMC Utrecht to ensure compliance with legislation and regulations (reference number 24U-1930).

## Funding

No specific funding was received for this work. ECRAID-Prime is funded by the European Union’s HORIZON 2021-2027 Research and Innovation Program (grant no. 101046109). In the EU, RECOVERY's assessment of treatments for flu is funded by Flu Lab. RECLAIM is funded by ZonMw (grant no. 11080012310002 and 11080022420007) and Stichting Long Covid. REMAP-CAP is funded by the EU Horizon 2020 Research and Innovation Program (grant nos. 965313, 602525, and 101003589), ZonMw (grant no. 10710022210003), the Health Research Board of Ireland (grant nos. CTN 2021-010, CTN 2014-012, and APRO-2023-017), the National Institute for Health and Care Research Imperial Biomedical Research Centre, the UK National Institute for Health and Care, and the French Ministry of Health (grant No. PHRC-20-0147). SNAP is funded by ZonMw (grant no. 10140022110014), the University Medical Center Utrecht, the Ministry of Science, Energy, Climate Protection and Environment (MWU) of the state of Saxony-Anhalt in Germany, the Network University Medicine (NUM)/Federal Ministry of Education and Research, the Swedish Research Council (grant no. 2023-06427), and the Stig and Ragna Gorthon's foundation (grant no. 2023-2916).

## Declaration of competing interest

The authors declare the following financial interests/personal relationships which may be considered as potential competing interests: During the conduct of this study, M.J.M.B. served as the CEO of Ecraid. Since January 1, 2026, L.P.G.D. is the CEO of Ecraid. Grants related to the trials included in this study are acknowledged under “funding”. In addition to grants or contracts, the authors declare the following financial interests/personal relationships which may be considered as potential competing interests: support for attending meetings and/or travel (A.J.d.J.), consulting fees (M.J.M.B.), participation on a Data Safety Monitoring Board or Advisory Board (M.J.M.B., L.P.G.D), leadership or fiduciary roles (M.J.M.B., L.P.G.D), and the receipt of trial medication (A.W.v.d.V. and L.P.G.D.). Completed disclosure of interests following the International Committee of Medical Journal Editors template are available from Appendix C.

## Data Availability

The datasets analyzed during the current study are no longer publicly available under the European Medicines Agency's revised transparency rules of 5 October 2023 (https://www.ema.europa.eu/en/documents/other/revised-ctis-transparency-rules_en.pdf). The manuscript text and practice-based recommendations (Appendix B) provide detailed information on the considerations and sponsor replies. Excerpts are available from the corresponding author on reasonable request.

## Glossary

COREQ: consolidated criteria for reporting qualitative research
CTIS: Clinical Trials Information System
CTR: Clinical Trials Regulation
DSMB: data safety monitoring board
EU: European Union
ICF: informed consent forms
IMP: investigational medicinal products
MREC: medical research ethics committee
NCA: national competent authority
NO: nitric oxide
RAR: response-adaptive randomization
RFI: requests for information

## Appendix A Supplementary data

Multimedia component 1 Short trial descriptions. Multimedia component 1

Multimedia component 2 Practice-based recommendations. Multimedia component 2

Multimedia component 3 Declaration of competing interests. Multimedia component 3
