# Supplementary material for: Considerations raised during the regulatory and ethics review of platform clinical trials in infectious diseases
Source: Contemp Clin Trials Commun. 2026 Mar 26;51:101633. doi: 10.1016/j.conctc.2026.101633 (PMC13054085; doi:10.1016/j.conctc.2026.101633)
Supplement: Multimedia component 1 — Short trial descriptions. [file mmc1.docx]

**Additional file 1. Short trial descriptions and trial characteristics**

**Box 1. Short trial descriptions.**

**ECRAID-Prime**

The European Clinical Research Alliance on Infectious Diseases – Primary care adaptive platform trial for pandemics and epidemics (ECRAID-Prime) investigates safety and efficacy of interventions in adult patients with COVID-19 or COVID-like-illness in the primary care setting, with a focus on early phase studies. A qualitative sub-study is conducted to understand experiences with the platform trial and acceptance of the medicinal products by healthcare professionals, researchers and patients.

**RECLAIM**

RECLAIM is an open-label platform trial that aims to determine the impact of repurposed drugs and devices on patient-reported health-related quality of life in adults with persistent post-acute sequelae of SARS-CoV-2 infection. Participation of pregnant women or those breastfeeding varies per intervention arms. RECLAIM is fully decentralized, with study activities implemented at participants’ homes, including video consultations for (pre-)screening, informed consent visits, eConsenting, online questionnaires, and direct-to-participant shipment of study medication.

**RECOVERY**

The Randomised Evaluation of COVID-19 ThERapY (RECOVERY) trial is an open-label trial that evaluates treatments for patients hospitalized for COVID-19, influenza, and community-acquired pneumonia (CAP) caused by other organisms. RECOVERY has been active in the UK since March 2020, and was initially designed to identify treatments for COVID-19, but was extended as a platform trial for CAP. Trial procedures are specifically designed to minimize site staff burden. In the EU, patients <18 years are not included.

**REMAP-CAP**

The Randomized Embedded Multifactorial Adaptive Platform trial for Community-Acquired Pneumonia (REMAP-CAP) evaluates the effectiveness of a range of interventions in multiple treatment domains in hospitalized patients with acute respiratory tract infection. All interventions in current domains are provided open-label. In the EU, only adults are included. REMAP-CAP has been ongoing since 2016 and was authorized under the EU Clinical Trials Directive (2001/20/EC). On 9 February 2024, the trial transitioned to the Clinical Trial Regulation (No 536/2014).

**SNAP**

The *Staphylococcus aureus* Network Adaptive Platform (SNAP) trial is an open-label platform trial addressing multiple therapeutic questions in patients with *S. aureus* bacteremia (SAB). Alternative to randomization in the domains of the SNAP trial, participants may participate in an observational registry. In the EU, no patients <18 years, pregnant women or breastfeeding women are included.

**Table 1. Trial characteristics**

| **Characteristic** | **Platform trial** | | | | |
| --- | --- | --- | --- | --- | --- |
| Trial acronym | **ECRAID-Prime** | **SNAP** | **RECLAIM** | **REMAP-CAP** | **RECOVERY** |
| EUCT number(s) | 2022-501707-27-00  2022-501707-27-01 | 2023-503582-35-00 | 2024-511580-28-00  2024-511580-28-01  2024-511580-28-02 | 2023-507889-89-00 | 2023-507441-29-00 |
| EU Sponsor | UMCU | UMCU | UMCU | UMCU | Oxford University |
| Trial phase | 2/3 | 4 | 3 | 2/3 | 4 |
| Medical condition | COVID-19 and COVID-like-illness | *Staphylococcus aureus* bacteremia | Post-acute sequelae of SARS-CoV-2 infection | Respiratory tract infection | Pneumonia |
| Low-intervention trial | Decided per compound | Yes | No | No | Yes |
| Reporting member state | Belgium | The Netherlands | The Netherlands | The Netherlands | The Netherlands |
| Investigational medicinal product | - Unauthorized IMP - nitric oxide nasal spray - saline nasal spray | - All IMPs are authorized - benzylpenicillin cefazolin - clindamycin - flucloxacillin - vancomycin | - All IMPs are authorized - colchicine - metformin | - All IMPs are authorized - amoxicillin-clavulanate - azithromycin - baloxavir marboxil - baricitinib - ceftriaxone - clarithromycin - dexamethasone - erythromycin - hydrocortisone - imatinib - levofloxacin - moxifloxacin - oseltamivir - piperacillin-tazobactam - roxithromycin - tocilizumab | - All IMPs are authorized - dexamethasone (or prednisolone or hydrocortisone in pregnant women) - oseltamivir |
| Number of sites in the EU/UK | - BE: 12 - DE: 1 - ES: 6 - FR: 1 - IE: 7 - PL: 6 - UK: 5 | - DE: 19 - NL: 10 - SE: 8 | - NL: 1 | - BE: 3 - CZ: 2 - DE: 11 - EE: 1 - ES: 11 - FR: 23 - HR: 3 - IE: 7 - IT: 10 - NL: 21 - PT: 2 - RO: 1 - SI: 2 - UK: 66 | - BE: 5 - EE: 4 - ES: 8 - FR: 12 - IT: 12 - NL: 3 - PT: 5 - RO: 7 - SE: 7 |
| Decision date^1^ | 8 April 2024 | 3 August 2023 | 7 February 2025 | 20 March 2024 | 2 February 2024 |
| Start date^2^ | 3 October 2024 | 26 October 2023 | 20 February 2025 | 19 February 2016 | 29 February 2024 |
| First patient first visit^3^ | 9 October 2024 | 26 October 2023 | 27 February 2025 | 11 April 2016 | 22 March 2024 |

Information presented for the countries managed by Ecraid available from the Clinical Trials Information System on 28 March 2025 ^1^ First decision date, date when the decision to authorize or not authorize a clinical trial application was made. ^2^ The first trial start date, the official date when the clinical trial begins in a Member State and is open for recruitment ^3^ First recruitment start date, the date when the first participant has been recruited (from https://www.ema.europa.eu/en/documents/other/clinical-trial-information-system-ctis-public-portal-summary_en.pdf). EU, European Union; IMP, investigational medicinal product; UMCU, University Medical Center Utrecht. Country abbreviations: BE, Belgium; CZ, Czech Republic; DE, Germany; EE, Estonia; ES, Spain; FR, France; HR, Croatia; IE, Ireland; IT, Italy; NL, the Netherlands; PT, Portugal; RO, Romania; SE, Sweden; SI, Slovenia; UK, United Kingdom.
