# Supplementary material for: Considerations raised during the regulatory and ethics review of platform clinical trials in infectious diseases
Source: Contemp Clin Trials Commun. 2026 Mar 26;51:101633. doi: 10.1016/j.conctc.2026.101633 (PMC13054085; doi:10.1016/j.conctc.2026.101633)
Supplement: Multimedia component 2 — Practice-based recommendations. [file mmc2.docx]

**Additional file 2.** Key recommendations based on considerations raised during the regulatory review of five infectious diseases platform trials.

| **Theme** | **Sub-theme** | **Generic item** | **Platform-specific item** |
| --- | --- | --- | --- |
| Clinical trial application | Regulatory management |  | - If applicable, provide a justification for why the platform trial evaluating a medical device is submitted under the CTR. When medical devices are evaluated, CE-certification documentation should be provided. - If applicable, provide a justification why the noninterventional registry is submitted under the CTR. It may differ per MSC whether an observational component of the trial is evaluated under the CTR or requires separate evaluation. |
|  | Submission requirements | - Ensure the submitted dossier is complete.^[[1]](#footnote-1)^ - Ensure all documents are named according to the [naming convention guidelines](https://www.hma.eu/fileadmin/dateien/HMA_joint/00-_About_HMA/03-Working_Groups/CTCG/2023_04_CTCG_Best_practice_guide_naming_of_documents__version_2.0.pdf). - Comply with language requirements. Review texts written in a local language to circumvent translation issues. Reference was made to [European Commission Q&A document](https://health.ec.europa.eu/document/download/bd165522-8acf-433a-9ab1-d7dceae58112_en?filename=regulation5362014_qa_en.pdf) Annex 2. - Be aware of different documentation requirements across MSCs (e.g., whether GCP certificates need to be uploaded to CTIS or whether detailing GCP training in a CV suffices). - Do not submit documents that will not be used in the respective MSC (e.g., recruitment arrangements). Reference was made to Q1.24 of the [European Commission Q&A document](https://health.ec.europa.eu/document/download/bd165522-8acf-433a-9ab1-d7dceae58112_en?filename=regulation5362014_qa_en.pdf) and [the preparation of the Research dossier Part I section on the CCMO website](https://english.ccmo.nl/investigators/clinical-trials-with-medicinal-products-ctr/preparation-ctr/research-dossier-part-i/protocol). - Ensure the payments to authorities are made in the correct currency. - Ensure consistency across trial-related documents and CTIS structured text fields (e.g., planned number of participants and sites, dates, version numbers, and document titles). - Ensure consistency between trial-related documents and responses to the RFIs. - Be aware that payments to multiple authorities may be required (e.g., national competent authority, national clinical trial compensation fund, national ethics committee in Poland).^[[2]](#footnote-2)^ - In the cover letter, provide information on whether the trial is a low-intervention trial, whether it is a first-in-human trial, the regulatory status of the IMPs, a statement on the CE marking of the medical device used, and the location of the reference safety information (i.e., section in the IB or SmPC). - Indicate the non-commercial nature of the trial in the cover letter as specific fee regulations are available for non-commercial trials in some MSCs. - In case an authorized product is modified (e.g., re-labelling), a simplified IMPD should be submitted. Reference was made to the [*Guideline on the requirements to the chemical and pharmaceuticals quality documentation concerning investigational medicinal products in clinical trials*](https://www.ema.europa.eu/en/documents/scientific-guideline/guideline-requirements-chemical-and-pharmaceutical-quality-documentation-concerning-investigational-medicinal-products-clinical-trials-revision-2_en.pdf). - For authorized IMP, explore the possibility to use the IMP’s authorized label instead of trial re-labelling. A justification for this approach should be provided. - CTIS technicalities may impede the upload of missing/requested documents (e.g., because of an ongoing substantial modification or incorrect lapse of the RFIs). Discuss CTIS issues with the MSCs. Reference was also made to the [EMA CTIS training modules](https://www.ema.europa.eu/en/human-regulatory-overview/research-development/clinical-trials-human-medicines/clinical-trials-information-system-ctis-training-support/clinical-trials-information-system-ctis-online-training-modules). - Provide definitions of terms such as “WOCBP” and “highly effective contraceptives” and circumvent subjective wording (e.g., “[the IMP] has an extremely high benefit to risk ratio”. - Review the terminology that is used. For example, refer to substantial modification instead of amendment, refer to anonymization and pseudonymization as appropriate and do not use words such as “pseudo-anonymization”, refer to legally authorized representative instead of legal representative, distinguish incapacity of will and incapacity of act, and distinguish severity and seriousness. Reference was made to Q7.7 of the [European Commission Q&A document](https://health.ec.europa.eu/document/download/bd165522-8acf-433a-9ab1-d7dceae58112_en?filename=regulation5362014_qa_en.pdf). - When documents are signed (e.g., CVs, compliance with use of biological samples document, SSA, DOI), ensure the documents are uploaded in the correct, searchable format – the original PDF/Word versions (instead of a scanned version) are uploaded to ensure accessibility. It was mentioned that these documents do not need to be signed, when a signed copy (of high quality when scanned) was already uploaded. - When changes are made to the documents, ensure to upload a clean and track changes version. The track changes versions should always be uploaded as “not for publication”. Make sure to have _Track_change/TC within the title so it is clear which documents are the track change versions. - If changes are made to the application, provide a List of Changes to the Application. In this regard, reference was made to the [“RFI Response List of Changes to the Application” document on the CTCG website](https://www.hma.eu/about-hma/working-groups/clinical-trials-coordination-group.html). Documents that have not been updated do not need to be mentioned in the modification description. - For trial-related documents that contain personally identifiable information or commercial confidential information (e.g., SSA forms, proof of insurance, cover letter, CVs, manufacturing and importation authorization), make sure to upload two identical documents, one of which redacted for publication to ensure compliance with the GDPR. Reference was made to [CTIS training modules 12 & 19](https://www.ema.europa.eu/en/human-regulatory-overview/research-development/clinical-trials-human-medicines/clinical-trials-information-system-ctis-training-support/clinical-trials-information-system-ctis-online-training-modules) and [EMA disclosure rules](https://www.ema.europa.eu/en/documents/other/revised-ctis-transparency-rules_en.pdf) and a [guidance document](https://accelerating-clinical-trials.europa.eu/document/download/6a0b836f-4779-4bb9-9584-1ce504a9ae38_en?filename=guidance-document-how-approach-protection-personal-data-commercially-confidential-information-while_.pdf). - The latest version of European Commission templates for application documents should be used (available from <https://health.ec.europa.eu/medicinal-products/eudralex/eudralex-volume-10_en>). - For some documents of national concern (e.g., those related to proof of payment, national requirements on data protection), national templates are available. Reference was made to the [Belgian ICF template](https://overlegorganen.gezondheid.belgie.be/nl/ICF-template-interv-trial-adult-patient-NL), [BAREC ICF template](https://barec.be/icf-template-interventional-clinical-trials-with-imp-on-adult-patientsctr-studies/), [AKEK ICF template](https://www.akek.de/wp-content/uploads/MUSTERTEXT_CTR_Info-Einwilligung-Patienten_Version-1.2.docx), [AKEK ICF biomaterial](https://www.akek.de/wp-content/uploads/Mustertext-AMG-akzessorische-Probensammlungen-2022-1.docx), [NREC templates](https://www.nrecoffice.ie/submit-under-the-clinical-trial-regulation/), [AEMPS proof of payment instructions](https://www.aemps.gob.es/investigacionClinica/medicamentos/docs/Instrucciones-v18-junio-2023.pdf), [CCMO cover letter template](https://english.ccmo.nl/investigators/clinical-trials-with-medicinal-products-ctr/preparation-ctr/form-and-msc/cover-letter), [CCMO protocol synopsis template](https://english.ccmo.nl/investigators/clinical-trials-with-medicinal-products-ctr/preparation-ctr/research-dossier-part-i/protocol), [CCMO proof of payment form](https://english.ccmo.nl/investigators/clinical-trials-with-medicinal-products-ctr/preparation-ctr/sections-form-and-msc/proof-of-payment-of-fee), [form for additional information clinical trials in France](https://sante.gouv.fr/IMG/docx/document_additionnel_ctr1.docx), [Läkemedelsverket proof of payment form](https://www.lakemedelsverket.se/en/forms/invoice-documentation-for-clinical-trial-application), [AIFA National Template for financial agreement](https://www.aifa.gov.it/-/assolvimento-dell-imposta-di-bollo-sulle-domande-di-autorizzazione-di-sperimentazioni-cliniche-e-modifiche-sostanziali), CCMO ICF template, and FAHMP SSA template. | - In a platform trial, the trial title may relate to the nature of the trial and not the comparisons. Consider adding a subtitle to explain the purpose of the study. - Update the insurance policy accordingly when arms are dropped or added. Additionally, the broad insurance coverage should be explained; to account for dropouts and a perpetual design and when the duration of the trial is not known beforehand. Ensure the insurance certificate is updated on time. - Make sure the overview of the approved domain-specific appendices in the cover letter is up-to-date when the platform trial includes multiple domains (and version numbers) to ensure MSCs have the correct oversight. - Ensure appendices are consistently and clearly referred to, because these are stand-alone documents. - The modular protocol approach with various appendices may be perceived as complex. It is recommended to clearly report on the protocol structure with as few appendices as possible. |
| Recruitment and informed consent | Arrangements for recruitment of trial participants | - Specify whom will obtain informed consent and that the eligibility assessment is performed by a physician. - In a trial with DCT elements, detail whether the GP will be involved in the recruitment as an investigator and the location where the ICF is signed, justify the remote screening approach or include an in-person screening visits to confirm participant eligibility. - Specify the exact amount for reimbursing participants and explain the process of reimbursement, including a rationale for the amount, and whether it is foreseen to reimburse any obligatory contraception. |  |
|  | Requirements informed consent | - Explain procedures that are different from routine care (including randomization) and explain when the interventions deviate or are in line with clinical guidelines. Additionally, provide information about alternative procedure(s) or course(s) of treatment that may be available to the subject, and related potential benefits and risks. - Provide information on the duration of participation, duration of the trial, expected number of participants, instructions on how data will be collected and by whom, and how data will be protected (confidentiality measures, data storage duration, location biological sample data storage, legal basis for data processing, and whom has access). - Include a statement that data protection may not be similar outside the EU when data is transferred to third countries. - Detail the characteristics of the IMP (in lay language), including the route of administration, when the IMP will be initiated, frequency of IMP administration, duration of the IMP use, indicate that the IMP will be provided free of charge, whether the IMP has a marketing authorization, contraindicated medications, and potential risks associated with IMP use (in line with the SmPC). - Explain how trial results will be communicated with participants. Provide the summary of the results (in lay language) within one year after completion of the clinical trial on <https://euclinicaltrials.eu/> and other registers when applicable. - Provide information about post-trial considerations (e.g., what happens if a participant withdraws or is withdrawn in terms of data use and safety follow-up; what post-trail treatment options are available; what follow-up measures are in place in case of premature clinical trial discontinuation) - Describe the purpose of (potential) secondary use of data (e.g., additional analysis, validation of diagnostic assays, specific medicine development). - Consent for future use of samples should be optional (i.e., separate consent should be obtained for this purpose) and indicate that MREC approval is needed for these future studies. - Describe and justify deferred consent procedures or consent by a LAR. Relatedly, explain that removing collected data of those who do not regain capacity to consent, may undermine the trial integrity. - If eConsent is used, consider providing written consent using paper consent forms, if the participant prefers this, and allow for face-to-face informed consent discussions (e.g., videoconferencing if consent is obtained remotely). Ensure and detail compliance with data protection requirements and governance arrangements of eConsent systems. | - Describe that a new ICF will be provided when interventions are added/removed. - Explain what a platform trial is and whether participants are free to choose to participate in domains, but that allocation to treatment arms within domains is random. |
| Participant safety and data protection | Data protection | - Specify whom will have access to personal data and specify the type of data, including the GP, persons responsible for quality control, commercial parties, researchers, external couriers for IMP shipment and sample pick up, and potential unauthorized access. - Confirm that the sponsor does not have access to patient identifying data and how this is ensured. - Provide details on biological sample storage (and whether biobank certificates are in place), online database (e.g., PRO), and data processing of those recruited via advertisement. - Assessors have a preference to analyze data within the EU, because the regulatory framework and data protection standards are known. Specify that data protection outside the EU is not guaranteed to be carried out in an equivalent manner, requires explicit consent, and describe how data will be protected (e.g., using standard contractual clauses). - Include a description of data breach measures (Regulation EU 536/2014, Annex 1, section D17)*.* - Specify data minimization measures (e.g., duration of retention – including when the retention period starts, data linkage procedures, justify the collection of race/ethnicity data, pseudonymization process). | - Data should be retained for 25 years (CTR) from the end of the study. In a platform trial, the study end may not be prespecified. Indicate when the 25 years start (e.g., end of the study or after a domain is closed, whichever comes first). |
|  | IMP characteristics | - Report on the contraindicated medications, describe the process of concomitant medication check, and list discontinuation criteria (i.e., AEs that will lead to discontinuation). - Specify the IMP safety profile and IMP-specific exclusion (e.g., renal impairment, required contraception measures in line with [CTCG guidance](https://www.hma.eu/fileadmin/dateien/Human_Medicines/01-About_HMA/Working_Groups/CTFG/2020_09_HMA_CTFG_Contraception_guidance_Version_1.1_updated.pdf)). - Provide a rationale for the IMP dose and duration and justify deviations from the SmPC – e.g., by referring to previous trials. - In trials with DCT elements, justify the approach to assess participant eligibility. | - Provide a rationale for the (future) IMP selection in the platform trial. |
|  | Safety management | - Provide a rationale for the inclusion or exclusion of pregnant and breastfeeding women and WOCBP, minors, incapacitated participants. - Include instructions on when participants should contact the investigative team in the intervention-specific appendix. - A reduced safety monitoring plan should be justified. In principle, all AEs should be collected until the end of study participation. Reference was made to [ICH E19](https://database.ich.org/sites/default/files/ICH_E19_Guideline_Step4_2022_0826_0.pdf) for the exceptions when the IMP has an established safety profile. - Specify the safety follow-up of participants who discontinue trial participation and distinguish between those who are withdrawn and those who withdraw consent. - Specify how SUSARs will be reported outside the EU (EudraVigilance database). - For trials with DCT elements, draft a contingency plan to minimize risks following the [Recommendation Paper on Decentralized Elements in Clinical Trials](https://health.ec.europa.eu/system/files/2023-03/mp_decentralised-elements_clinical-trials_rec_en.pdf). - Ensure the qualifications of investigative staff are clear and justified. | - Participation of vulnerable groups may differ between domains and countries, and a rationale should be provided. - Indicate that, if an IMP is added later in a platform trial and is evaluated in minors, this will be justified and is subject to NCA/MREC review. |
| Study design | Indication | - Specify any subgroup analyses, which may be considered important when the indication consists of heterogeneous presentations. | - In a platform trial, interventions may be evaluated in a broad indication. Clearly explain the indication (e.g., syndromic illness, potential heterogeneous population) under study. Relatedly, report more specific domain/intervention-specific eligibility criteria. |
|  | Blinding and comparisons | - Provide a rationale for an open-label setup. | - Clearly describe the control arm for the primary analysis, particularly when multiple comparisons are possible in a platform trial design. |
|  | Data collection | - Consider the provision of paper diaries to those individuals who may not be able to complete online questionnaires. - Justify data collection by participants themselves (e.g., self-swabbing) and how data quality will be ensured – e.g., by referring to previous studies, providing clear instructions, and whether the endpoint is a primary/secondary endpoint. - Describe how participants will be trained on data collection procedures. |  |
|  | Appropriateness platform design |  | - Justify the platform design, when – at the start of the trial – only one intervention is evaluated. - Ensure a good trial infrastructure to leverage the advantages of the adaptive platform design (e.g., complete and accurate data to enable high-quality interim analyses). |
|  | Endpoints | - Justify the use of endpoints that may be perceived as subjective (e.g., time to feeling recovered) by referring to the clinical relevance and expected event rate. | - Clearly explain different primary endpoints for different “phase type” evaluations in a platform trial. For each domain, clearly report the respective primary endpoint. |
|  | Statistical approaches | - Detail the adjustment for covariates and describe stratifications in randomization (minimization) and analysis. - Detail procedures to limit missing data and describe imputation strategies in the protocol and/or statistical analysis plan. | - Specify whether nonconcurrent controls are included in future domains. If nonconcurrent controls are included, consider concurrent controls as a sensitivity analysis. - Justify Bayesian approach with extensive simulations, justify the assumptions, and report on the results of the simulations (i.e., probabilities of falsely/correctly rejecting the null hypotheses). - Provide details on the planned interim analyses (i.e., frequency, outcomes, statistical model, correction for multiplicity, stopping rules). - Describe stopping criteria or justify why these are not prespecified. - Provide details on the sample size calculation and power. Include estimates of expected number of participants, to demonstrate the feasibility of achieving the target sample size. |

This table includes prioritized recommendations for clinical trial applications. Note that this table does not provide a full overview of all comments that were raised but rather provides a comprehensive overview in which the actionable comments are summarized. AE, adverse event; CV, curriculum vitae; CE, Conformité Européenne; CTCG, Clinical Trials Coordination Group; CTR, clinical trials regulation; DCT, decentralized clinical trial; DOI, declaration of interest; EU, European Union; GCP, good clinical practice; GDPR, general data protection regulation; GP, general practitioner; ICF, informed consent form; IMP(D), investigational medicinal product (dossier); LAR, legally authorized representative; NCA, national competent authority; MREC, medical research ethics committee; MSC, member state concerned; PRO, participant reported outcome; SmPC, summary of product characteristics; SSA, site suitability assessment; SUSAR, suspected unexpected serious adverse reaction; WOCBP, women of childbearing potential.

1. We found that the following documents were frequently requested by assessors: (a) CVs, including experience with clinical research and patient care, and a recent GCP certificate, (b) list of sites, (c) financial arrangements (amongst others, clinical trial agreements with sites, including non-financial agreements), (d) site suitability form, (e) proof of insurance, (f) lay language synopsis, (g) DSMB charter, (h) a copy of the manufacturing and import authorization, (i) a qualified person declaration, (j) recruitment and informed consent procedure document in local language, (k) a schedule of activities in the protocol including adverse event monitoring and assessment of concomitant medication activities, (l) a receipt of the data protection authority, (m) a statement of compliance with national requirements on data protection (in local language), (n) a statement from the electronic consent IT service provider, (o) immediate IMP label. [↑](#footnote-ref-1)
2. Of note, this seems to be in conflict with Article 87 of Regulation (EU) No 536/2014 which states that “A Member State shall not require, for an assessment as referred to in Chapters II and III, multiple payments to different bodies involved in this assessment”. [↑](#footnote-ref-2)
